# A Comparison Study of the Detection Limit of Omicron SARS-CoV-2 Nucleocapsid by Various Rapid Antigen Tests

**DOI:** 10.3390/bios12121083

**Published:** 2022-11-27

**Authors:** Daniela Dobrynin, Iryna Polischuk, Boaz Pokroy

**Affiliations:** 1 Department of Materials Science and Engineering, Technion—Israel Institute of Technology, Haifa 32000, Israel; 2 The Russel Berrie Nanotechnology Institute, Technion—Israel Institute of Technology, Haifa 32000, Israel

**Keywords:** rapid antigen test, SARS-CoV-2, COVID-19, omicron variant

## Abstract

Rapid antigen tests (RATs) are widely used worldwide to detect SARS-CoV-2 since they are an easy-to-use kit and offer rapid results. The RAT detects the presence of the nucleocapsid protein, which is located inside the virus. However, the sensitivity of the different RATs varies between commercially available kits. The test result might change due to various factors, such as the variant type, infection date, swab’s surface, the manner in which one performs the testing and the mucus components. Here, we compare the detection limit of seven commercially available RATs by introducing them to known SARS-CoV-2 nucleocapsid protein amounts from the Omicron variant. It allows us to determine the detection limit, disregarding the influences of other factors. A lower detection limit of the RAT is necessary since earlier detection will help reduce the spread of the virus and allow faster treatment, which might be crucial for the population at risk.

## 1. Introduction

Since the first case of COVID-19 disease in Wuhan in December 2019, there has been a worldwide struggle to reduce the transmission of acute respiratory syndrome coronavirus 2 (SARS-CoV-2). Many countries worldwide imposed temporary local lockdowns in order to reduce person-to-person interactions [1,2], masks became obligatory, especially in closed spaces [3,4], and there was a general requirement for social distancing. However, the most efficient method to reduce the continuing spread of infection among the population, and in the meantime maintain a regular daily life, is the early detection of contagious infected people.

An extensive body of research aims to develop new technologies for SARS-CoV-2 detection [5,6,7,8]. One known and reliable method is the reverse-transcriptase PCR test (RT-PCR), which is constantly improving [9]. It is possible to detect the virus even if there is only one RNA strand in the sample and run hundreds of samples simultaneously [10]. This method has a few disadvantages, such as a high cost, is time consuming, the need for medical laboratories and skilled staff to perform the test, and the major flaw: the lack of an appropriate number of available tests. The latterly prominent Omicron variant (B.1.1.529) and its derivatives have caused a tremendous increase in the number of infected people due to its enhanced transmissibility [11,12]. All the above points emphasize the high demand for easy-to-use, cheap, and available detection tests for SARS-CoV-2 and other pathogens [13].

The solution was found in developing rapid antigen tests (RATs), which are based on lateral flow immunoassay technology (LFIA) and provide a result within 15–30 min. For a more detailed description regarding the principles of LFIA, readers are referred to Koczula et al. [14]. The majority of industrially manufactured RATs are designed based on the detection of the nucleocapsid protein, one of four main structural proteins in the SARS-CoV-2 [15]. The nucleocapsid protein has proved to be a good diagnostic marker since it is detectable even after one day from the disease onset [16]. The key role of the nucleocapsid protein is to pack the viral genome into helical complexes named ribonucleocapsid (RNP), which interacts with the membrane protein (M) [17].

Former studies tested the RATs’ validity by examining PCR-positive COVID-19 patients samples or the isolated virus with various RATs [18,19,20,21,22]. These studies are very informative; however, many factors might affect the RAT result, such as the diversity in the viral load (VL, the virus amount in the sample) or the nucleocapsid concentration in each sample, the swab’s surface, exposure to different variants, date of infection, immune system reaction to the virus, vaccination status, etc. For instance, it was shown that various RATs tend to detect the Delta or Omicron variants at different sensitivities [21,23]. The latter implies that infected patients who show PCR-positive results might have a RAT-negative result simply due to each person’s uniqueness and situation. Moreover, the nasopharyngeal samples contain many other proteins which might affect the detection of the nucleocapsid protein. In previous studies, it was shown that there is a significant variation in sensitivity between different RATs when applied to both symptomatic participants (34.3–91.3%) and asymptomatic participants (28.6–77.8%) [24]. The sensitivity of RT-PCR tests is higher than that of the RAT tests [24,25]. Nevertheless, the lower the RAT’s detection limit, the earlier one can detect COVID-19-infected patients. This is important in cases when urgent treatment is needed, as well as to facilitate early isolation in order not to infect others.

This study compares seven commercially available RATs for detecting SARS-CoV-2 by a simple direct experiment. In contrast to previous studies, here we examined the various RATs by testing with various known, pure, and pre-defined concentrations of Omicron SARS-CoV-2 nucleocapsid protein solutions. The tests were performed according to each manufacturer’s instructions utilizing the RAT solution containing known volumes and concentrations of the nucleocapsid protein of the Omicron variant. This method allowed us to determine the detection limit of each RAT while controlling the amount of the added nucleocapsid protein and disregarding all other influencing factors.

## 2. Materials and Methods

The nucleocapsid protein of the Omicron variant was purchased from BPS Bioscience (San Diego, CA, USA) and contained the mutations: P13L, R203K, G204R, and ∆31-33ERSdel. We used seven commercially available antigen tests: (a) Deepblue COVID-19 (SARS-CoV-2) Antigen Test Kit (colloidal gold) (Anhui Deepblue Medical Technology Co., Ltd., Hefei, Anhui, China); (b) Easy Diagnosis COVID-19 (SARS-CoV-2) Antigen Test Kit (Wuhan EasyDiagnosis Biomedicine Co., Ltd., Wuhan, Hubei, China); (c) EcoTest COVID-19 TO-GO (Assure Tech. (Hangzhou) Co., Ltd., Hangzhou, China); (d) GenSure COVID-19 Antigen Rapid Test Kit (GenSure Biotech Inc., Shijiazhuang, Hebei, China); (e) Orient Gene Rapid COVID-19 (Antigen) Self-Test (Zhejiang Orient Gene Biotech Co., Ltd., Huzhou, Zhejiang, China); (f) Panbio COVID-19 Antigen Self-Test (Abbott Laboratories, Chicago, IL, USA); (g) YHLO GLINE-2019-nCoV Ag for self-testing (Shenzhen YHLO Biotech Co., Ltd., Shenzhen, China).

The protein was diluted in Phosphate buffer saline (PBS), pH 7.4 (Sigma-Aldrich, St. Louis, MO, USA) to various concentrations: 550; 55; 5.5; 2.75; 1; 0.55; and 0.275 µg mL^−1^. After use, the protein solutions were stored at −20 °C (for the experiments which were conducted in March and April 2022). For the experiments held in October 2022, new protein solutions were prepared in the same way as before (the same pipettes and volumes were used). The (f) RAT is not included in these experiments (RH 60%) since, by this time, the RAT was not marketing anymore. The highest concentration is 550 µg mL^−1^, similar to the concentration delivered by BPS Bioscience (580 µg mL^−1^). A total of 5 µL from the relevant nucleocapsid concentration solution was added to each RAT test solution with a pipette. There was no use of a swab. The volume of the various test solutions varies between the different companies (250–400 µL, Table S1), but the total protein amount present in each test solution is identical. The protein in the test solution was mixed by pipetting until the protein was homogeneously dissolved. At the next step, as described in each manufacturer’s instructions, a specific number of drops were dripped from the test solution to the designated sample loading location at the RAT. After 15 min, the tested RATs were examined by the naked eye, and images were acquired (Figures S1–S7 in Supplementary Materials).

Performance of all the RATs is based on the same concept; the C-line is the control line and should appear if the RAT was conducted correctly, and the T-line appears if the nucleocapsid protein was detected.

Each type of RAT was firstly checked with the highest nucleocapsid concentration, and if the result was clearly positive (the T-line was bold and clear), we proceeded to test lower concentrations. If the T-line was barely visible, we performed three RAT replicates and continued to lower the protein concentration. Once the T-line was completely not visible, we again tested three replicates to ensure a negative result. The detection limit was determined as the lowest protein concentration, where all three replicates show a positive result.

All experiments were conducted in the fume hood at 21 °C. The relative humidity (RH) was stabilized by dehumidifier SAD50 (SmartAir, Kfar Kasem, Israel) to 30% (March and April months) and 60% (October).

## 3. Results and Discussion

This work provides an evaluation of seven commercially available RATs widely used internationally. In order to determine and compare the detection limit of different RATs, we directly used Omicron nucleocapsid protein solutions in decreasing concentrations. The protein concentration was lowered continuously until only the control line (C-line) was visible, rather than the two lines (C- and T-lines). The same volume of the protein solution (5 µL) was added to the test solutions each time, implying that the total amount of the nucleocapsid was identical. The performance of the different RATs was further compared, eliminating any human factor, such as the manner in which one may conduct the RAT and the actual viral load of the tested patient. The experiments were conducted under different relative humidity (RH) conditions of 30% and 60%.

**Figure 1:**
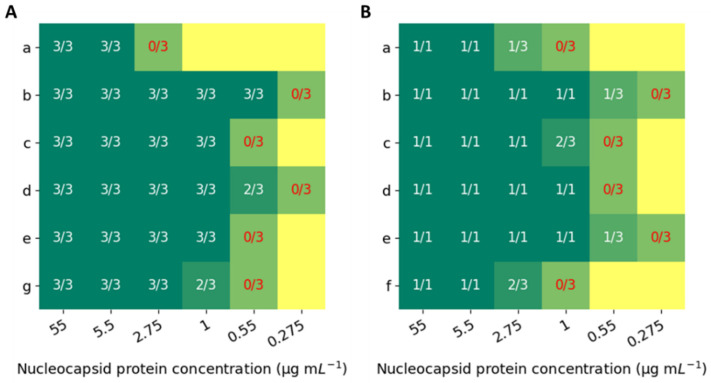
Various RATs were tested to detect a pure nucleocapsid protein in different concentrations, at RH conditions of (**A**) 60% and (**B**) 30%. The RATs are: (a) Deepblue COVID-19 (SARS-CoV-2) Antigen Test Kit (colloidal gold); (b) Easy Diagnosis COVID-19 (SARS-CoV-2) Antigen Test Kit; (c) EcoTest COVID-19 TO-GO; (d) GenSure COVID-19 Antigen Rapid Test Kit; (e) Orient Gene Rapid COVID-19 (Antigen) Self-Test; (f) Panbio COVID-19 Antigen Self-Test; (g) YHLO GLINE-2019-nCoV Ag for self-testing. Within each square are presented a number of positive results out of the replicates’ number.

Our results suggest that all RATs were able to detect the nucleocapsid protein using the 5.5 µg mL^−1^ solution, while the lowest detected concentration was 0.55 µg mL^−1^ by (b) RAT (Figure 1). In general, the reduction in the concentration of nucleocapsid protein caused the T-line to be weaker and thinner, as expected. At RH 60%, (c), (d), and (e) RATs were able to detect the nucleocapsid until 1 µg mL^−1^ solution. (g) RAT showed positive results until 2.75 µg mL^−1^ nucleocapsid concentration. These results provide additional evidence to previous studies suggesting that there might be significant variation between different RATs in detecting SARS-CoV-2, especially the Omicron variant. In this study, we observe up to one order of magnitude of nucleocapsid concentration difference between the several RATs. A reduced sensitivity was observed at RH 30% compared to RH 60% for the same protein concentration. For instance, for (b) RAT at RH 30%, only one out of three replicates showed a positive result at a concentration of 0.55 µg mL^−1^, while at RH 60% all replicates showed a clear T-line. In the case of the (c) and (d) RATs, the results are improved as well; for (c) RAT, all replicates showed positive results in RH 60% (1 µg mL^−1^), while in RH 30%, only two out of three. The (d) RAT could not detect the protein in 0.55 µg mL^−1^ concentration in RH 30%, in contrast to RH 60%, where two out of three replicates showed a T-line. A previous study showed that at an RH higher than 60% the water evaporation is reduced, and the diffusion across the nitrocellulose strip is improved, which obtains the desired signal [26]. The different results in the same RATs might be due to inaccuracies in the fabrication process or storage and shipment conditions.

There might be several explanations for the change in the detection limit between the different RATs, which are mainly attributed to variations in the manufacturing process. For instance, the T-line color stems from various sources, in particular either colloidal gold (RATs (a), (b), (g)), color conjugation (RAT(c)), polymer label (RAT (d)), etc. Additionally, the development process of the anti-nucleocapsid antibodies might vary between the companies, which would cause the recognition of different epitopes with different affinity and specificity. Moreover, the number of conjugated antibodies at the T-line location might also affect the detection limit. The latter might be the cause for achieving different results under the same conditions (same RAT, concentration, and RH).

As seen in Figure S8, we compared three different RATs using the same nucleocapsid concentration of the nucleocapsid protein. The emerged T-lines are not similar; in the case of RAT (f), the T-line is very thick and clear, the T-line observed on RAT (d) is weaker but detectable by the human eye, and in the case of RAT (a), the T-line is the least prominent. On the other hand, RAT (d) shows a better detection capacity than these two RATs.

Previous studies have shown that all twelve nucleocapsid proteins interact with ~800 nucleotides of genomic RNA to produce an RNP complex [27]. Within each virus, there are 35–40 RNPs, meaning that the total amount of nucleocapsid protein varies between 420 and 480 entities according to this model. Assuming that the weight of the protein is 48 kDa, we can translate the amount of protein to the corresponding number of viruses. For instance, 5 µL of 55 µg mL^−1^ nucleocapsid solution is equivalent to a protein mass of 275 ng, which translates to 7.19–8.21 × 10^9^ virions. The lowest detectable protein concentration of 0.55 µg mL^−1^ corresponds to 7.19–8.21 × 10^7^ virions. This analysis emphasizes the high amount of virus copies needed to receive a positive result, in contrast to nucleic acid amplification techniques, which can detect an infected person in the presence of only several virions.

The average VL of nasopharyngeal swabs (NS) in infected patients is about ~10^8^ genome copies per mL, and it varies between 10^5^–10^12^ genome copies per mL [28,29]. Such high variation is possible due to various factors, such as vaccination, variant type, number of days since infection, etc. For instance, the VL of vaccinated patients is lower than that of non-vaccinated patients, and the Delta variant causes a higher VL than that of the Omicron variant [29]. Thus, it is reasonable that a higher VL will lead to a higher amount of the nucleocapsid protein in the test solution and a lower probability of having a false-negative result.

Our study has several limitations. We have consistently added a specific volume of the protein solution to the test solution. In contrast, during real-life testing, the nasal mucus that discharges to the test solution is not identical and might be higher or lower. Moreover, here we use pure nucleocapsid protein dissolved in PBS, while the nasal samples contain the whole virus, and the nucleocapsid interacts with the RNA and M protein of the virus. This might cause the nucleocapsid epitopes to be unavailable for interaction with the RAT’s antibodies and reduce the detection efficiency. Furthermore, the nasal samples contain various additional proteins, such as mucin, and even other microorganisms. The factors mentioned above might cause changes in the interaction of the nucleocapsid protein with the antibodies present in the RAT, in contrast to the pure nucleocapsid we used. However, the experimental conditions are identical for all the tested RATs, which allows a reliable comparison. In addition, as we can see in Figures S1–S7, for the lowest detectable protein concentration, in some cases only, one out of three replicates showed a positive result. This means there are also variations in performance between the RATs from the same company. The increased number of tested RATs of each type may provide an even more reliable determination of the detection limits. However, the question arises whether the patient will persist and perform the test in a real-life situation more than a few times.

## 4. Conclusions

Our study demonstrates a simple method to evaluate the detection limit of various RATs using known pre-defined amounts of nucleocapsid protein. Here, as previously shown in other studies, yet in a new manner, there is a noticeable difference in the detection limits of various commercially available RATs, which customers and medical workers need to take into account when performing the RAT. We found the nucleocapsid protein mass of the various commercial RATs in the range of 2.75–27.5 ng in a test solution to be the lower detection limit for the RATs. This protein mass correlates to protein concentrations in the test solutions ranging between 9.8–78.6 ng mL^−1^. Obviously, this demonstrates not only the detection limit of RATs in general but also the variation in detection limits between various commercial RATs (eight-fold). Using a pure recombinant protein for RATs’ assessment is a simple and cheap method and does not require performing tests on patients (i.e., avoiding the need for a Helsinki declaration) or preserving their samples. For future use, if a new variant of concern emerges, for a quick assessment, it would not be necessary to wait for infected patients in order to test the RATs on human samples, but rather study the updated recombinant protein and compare it to previous ones. Additionally, it will allow the RATs producers to evaluate whether any change is needed in previously marketed RATs, to obtain more reliable tests.

## Data Availability

Not applicable.

## Supplementary Materials

The following supporting information can be downloaded at: https://www.mdpi.com/article/10.3390/bios12121083/s1, Table S1. Volumes of test solutions of various rapid antigen tests; Figure S1. “Deepblue COVID-19 (SARS-CoV-2) Antigen Test Kit (colloidal gold)” RATs after reaction with different concentrations of nucleocapsid protein at RH conditions of (A) 30% and (B) 60%. The lowest concentration that showed a positive result is 2.75 µg mL^−1^; only one out of three RATs showed a weak T-line (red arrow) at RH 30%; Figure S2. “Easy Diagnosis COVID-19 (SARS-CoV-2) Antigen Test Kit” RATs after reaction with different concentrations of nucleocapsid protein at RH conditions of (A) 30% and (B) 60%. The lowest concentration that showed positive result, in all replicates at RH 60%, is 0.55 µg mL^−1^; only one out of three RATs showed a weak T-line (red arrow) at RH 30%; Figure S3. “EcoTest COVID-19 TO-GO” RATs after reaction with different concentrations of nucleocapsid protein. The lowest concentration that showed positive result is 1 µg mL^−1^ at RH conditions of (A) 30% and (B) 60%; Two out of three RATs showed a weak T-line (red arrows) at RH 30% and all replicates at 60%; Figure S4. “GenSure COVID-19 Antigen Rapid Test Kit” RATs after reaction with different concentrations of nucleocapsid protein at RH conditions of (A) 30% and (B) 60%. The lowest concentration that showed positive result is 0.55 µg mL^−1^ at RH 60%; two out of three tests showed a positive result (red arrows); Figure S5. “Orient Gene Rapid COVID-19 (Antigen) Self-Test” RATs after reaction with different concentrations of nucleocapsid protein at RH conditions of (A) 30% and (B) 60%. The lowest concentration that showed a positive result is 1 µg mL^−1^ at RH 60%. Only one out of three RATs showed a weak T-line (red arrow) using a concentration of 0.55 µg mL^−1^, RH 30%; Figure S6. “Panbio COVID-19 Antigen Self-Test” RATs after reaction with different concentrations of nucleocapsid protein at RH 30%. The lowest concentration that showed positive result is 2.75 µg mL^−1^; Two out of three RATs showed a T-line (red arrows); Figure S7. “YHLO GLINE-2019-nCoV Ag for self-testing” RATs after reaction with different concentrations of nucleocapsid protein at RH 60%. The lowest concentration that showed positive result is 1 µg mL^−1^; Two out of three RATs showed a weak T-line (red arrow); Figure S8. Three different RATs after reaction with 5.5 µg mL^-1^ nucleocapsid concentration on the same day. The T-line is the least prominent in the case of “Deepblue COVID-19 (SARS-CoV-2) Antigen Test Kit (colloidal gold)” RAT, intermediate intensity of the T-line observed in the case of “GenSure COVID-19 Antigen Rapid Test Kit” RAT, and the “Panbio COVID-19 Antigen Self-Test” RAT showed the most prominent T-line.

## References

[1] The Lancet (2020). India under COVID-19 Lockdown. Lancet.

[2] Di Domenico L., Pullano G., Sabbatini C.E., Boëlle P.-Y., Colizza V. (2020). Impact of Lockdown on COVID-19 Epidemic in Île-de-France and Possible Exit Strategies. BMC Med..

[3] Feng S., Shen C., Xia N., Song W., Fan M., Cowling B.J. (2020). Rational Use of Face Masks in the COVID-19 Pandemic. Lancet Respir. Med..

[4] Chua M.H., Cheng W., Goh S.S., Kong J., Li B., Lim J.Y.C., Mao L., Wang S., Xue K., Yang L. (2020). Face Masks in the New COVID-19 Normal: Materials, Testing, and Perspectives. Research.

[5] Erdem Ö., Eş I., Saylan Y., Inci F. (2022). Unifying the Efforts of Medicine, Chemistry, and Engineering in Biosensing Technologies to Tackle the Challenges of the COVID-19 Pandemic. Anal. Chem..

[6] Huergo M.A.C., Thanh N.T.K. (2021). Current Advances in the Detection of COVID-19 and Evaluation of the Humoral Response. Analyst.

[7] Thapa S., Singh K.R., Verma R., Singh J., Singh R.P. (2022). State-of-the-Art Smart and Intelligent Nanobiosensors for SARS-CoV-2 Diagnosis. Biosensors.

[8] Asghar R., Rasheed M., ul Hassan J., Rafique M., Khan M., Deng Y. (2022). Advancements in Testing Strategies for COVID-19. Biosensors.

[9] Wang J., Cai K., Zhang R., He X., Shen X., Liu J., Xu J., Qiu F., Lei W., Wang J. (2020). Novel One-Step Single-Tube Nested Quantitative Real-Time PCR Assay for Highly Sensitive Detection of SARS-CoV-2. Anal. Chem..

[10] Guglielmi G. (2020). The Explosion of New Coronavirus Tests That Could Help to End the Pandemic. Nature.

[11] Chen J., Wang R., Gilby N.B., Wei G.-W. (2022). Omicron Variant (B.1.1.529): Infectivity, Vaccine Breakthrough, and Antibody Resistance. J. Chem. Inf. Model..

[12] Poudel S., Ishak A., Perez-Fernandez J., Garcia E., León-Figueroa D.A., Romaní L., Bonilla-Aldana D.K., Rodriguez-Morales A.J. (2022). Highly Mutated SARS-CoV-2 Omicron Variant Sparks Significant Concern among Global Experts—What Is Known so Far?. Travel Med. Infect. Dis..

[13] Castillo-León J., Trebbien R., Castillo J.J., Svendsen W.E. (2021). Commercially Available Rapid Diagnostic Tests for the Detection of High Priority Pathogens: Status and Challenges. Analyst.

[14] Koczula K.M., Gallotta A. (2016). Lateral Flow Assays. Essays Biochem..

[15] Bai Z., Cao Y., Liu W., Li J. (2021). The SARS-CoV-2 Nucleocapsid Protein and Its Role in Viral Structure, Biological Functions, and a Potential Target for Drug or Vaccine Mitigation. Viruses.

[16] Che X.-Y., Hao W., Wang Y., Di B., Yin K., Xu Y.-C., Feng C.-S., Wan Z.-Y., Cheng V.C.C., Yuen K.-Y. (2004). Nucleocapsid Protein as Early Diagnostic Marker for SARS. Emerg. Infect. Dis..

[17] Lu S., Ye Q., Singh D., Cao Y., Diedrich J.K., Yates J.R., Villa E., Cleveland D.W., Corbett K.D. (2021). The SARS-CoV-2 Nucleocapsid Phosphoprotein Forms Mutually Exclusive Condensates with RNA and the Membrane-Associated M Protein. Nat. Commun..

[18] Diao B., Wen K., Zhang J., Chen J., Han C., Chen Y., Wang S., Deng G., Zhou H., Wu Y. (2021). Accuracy of a Nucleocapsid Protein Antigen Rapid Test in the Diagnosis of SARS-CoV-2 Infection. Clin. Microbiol. Infect..

[19] Mak G.C., Cheng P.K., Lau S.S., Wong K.K., Lau C., Lam E.T., Chan R.C., Tsang D.N. (2020). Evaluation of Rapid Antigen Test for Detection of SARS-CoV-2 Virus. J. Clin. Virol..

[20] Porte L., Legarraga P., Vollrath V., Aguilera X., Munita J.M., Araos R., Pizarro G., Vial P., Iruretagoyena M., Dittrich S. (2020). Evaluation of a Novel Antigen-Based Rapid Detection Test for the Diagnosis of SARS-CoV-2 in Respiratory Samples. Int. J. Infect. Dis..

[21] Osterman A., Badell I., Basara E., Stern M., Kriesel F., Eletreby M., Öztan G.N., Huber M., Autenrieth H., Knabe R. (2022). Impaired Detection of Omicron by SARS-CoV-2 Rapid Antigen Tests. Med. Microbiol. Immunol..

[22] Cubas-Atienzar A.I., Kontogianni K., Edwards T., Wooding D., Buist K., Thompson C.R., Williams C.T., Patterson E.I., Hughes G.L., Baldwin L. (2021). Limit of Detection in Different Matrices of 19 Commercially Available Rapid Antigen Tests for the Detection of SARS-CoV-2. Sci. Rep..

[23] Stanley S., Hamel D.J., Wolf I.D., Riedel S., Dutta S., Contreras E., Callahan C.J., Cheng A., Arnaout R., Kirby J.E. (2022). Limit of Detection for Rapid Antigen Testing of the SARS-CoV-2 Omicron and Delta Variants of Concern Using Live-Virus Culture. J. Clin. Microbiol..

[24] Dinnes J., Sharma P., Berhane S., van Wyk S.S., Nyaaba N., Domen J., Taylor M., Cunningham J., Davenport C., Dittrich S. (2022). Rapid, Point-of-Care Antigen Tests for Diagnosis of SARS-CoV-2 Infection. Cochrane Database Syst. Rev..

[25] Chu V.T., Schwartz N.G., Donnelly M.A.P., Chuey M.R., Soto R., Yousaf A.R., Schmitt-Matzen E.N., Sleweon S., Ruffin J., Thornburg N. (2022). Comparison of Home Antigen Testing with RT-PCR and Viral Culture during the Course of SARS-CoV-2 Infection. JAMA Intern. Med..

[26] Choi J.R., Hu J., Feng S., Wan Abas W.A.B., Pingguan-Murphy B., Xu F. (2016). Sensitive Biomolecule Detection in Lateral Flow Assay with a Portable Temperature–Humidity Control Device. Biosens. Bioelectron..

[27] Klein S., Cortese M., Winter S.L., Wachsmuth-Melm M., Neufeldt C.J., Cerikan B., Stanifer M.L., Boulant S., Bartenschlager R., Chlanda P. (2020). SARS-CoV-2 Structure and Replication Characterized by in Situ Cryo-Electron Tomography. Nat. Commun..

[28] Singanayagam A., Hakki S., Dunning J., Madon K.J., Crone M.A., Koycheva A., Derqui-Fernandez N., Barnett J.L., Whitfield M.G., Varro R. (2022). Community Transmission and Viral Load Kinetics of the SARS-CoV-2 Delta (B.1.617.2) Variant in Vaccinated and Unvaccinated Individuals in the UK: A Prospective, Longitudinal, Cohort Study. Lancet Infect. Dis..

[29] Puhach O., Adea K., Hulo N., Sattonnet P., Genecand C., Iten A., Bausch F.J., Kaiser L., Vetter P., Eckerle I. (2022). Infectious Viral Load in Unvaccinated and Vaccinated Individuals Infected with Ancestral, Delta or Omicron SARS-CoV-2. Nat. Med..

