# Supplementary material for: A Comparison Study of the Detection Limit of Omicron SARS-CoV-2 Nucleocapsid by Various Rapid Antigen Tests
Source: Biosensors (Basel). 2022 Nov 27;12(12):1083. doi: 10.3390/bios12121083 (PMC9775131; doi:10.3390/bios12121083)
Supplement: Supplementary file 1 [file biosensors-12-01083-s001.zip › biosensors-2022090-supplementary.pdf]

Supplementary Data

Table S1. Volumes of test solutions of various rapid antigen tests.

RAT	Test Solution Volume (μL)
a	350
b	280
c	300
d	250
e	250
f	400
g	300

A

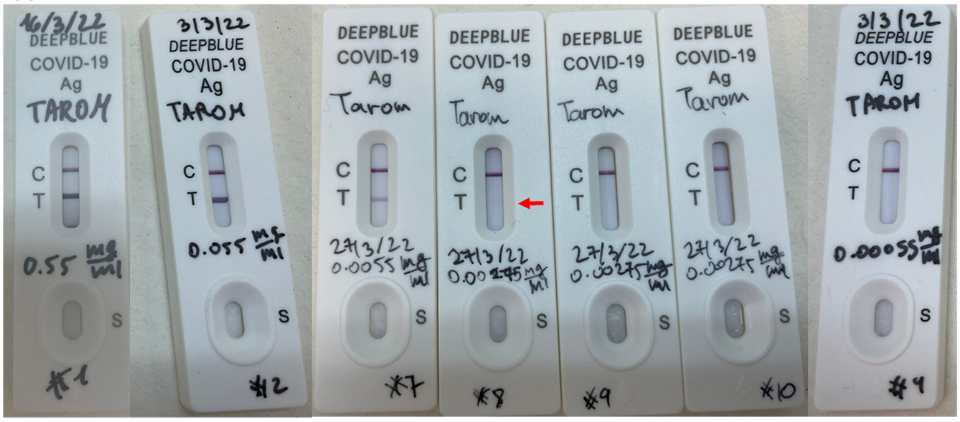

B

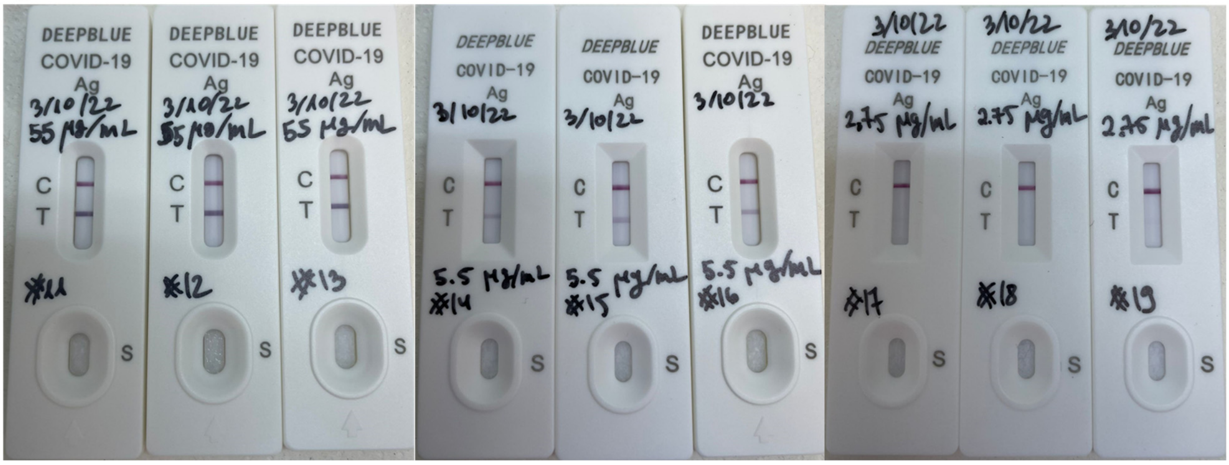

Figure S1. "Deepblue COVID-19 (SARS-CoV-2) Antigen Test Kit (colloidal gold)" RATs after reaction with different concentrations of nucleocapsid protein at RH conditions of (**A**) 30% and (**B**) 60%. The lowest concentration that showed positive result is 2.75 μg mL^{-1}; only one out of three RATs showed a weak T-line (red arrow) at RH 30%.

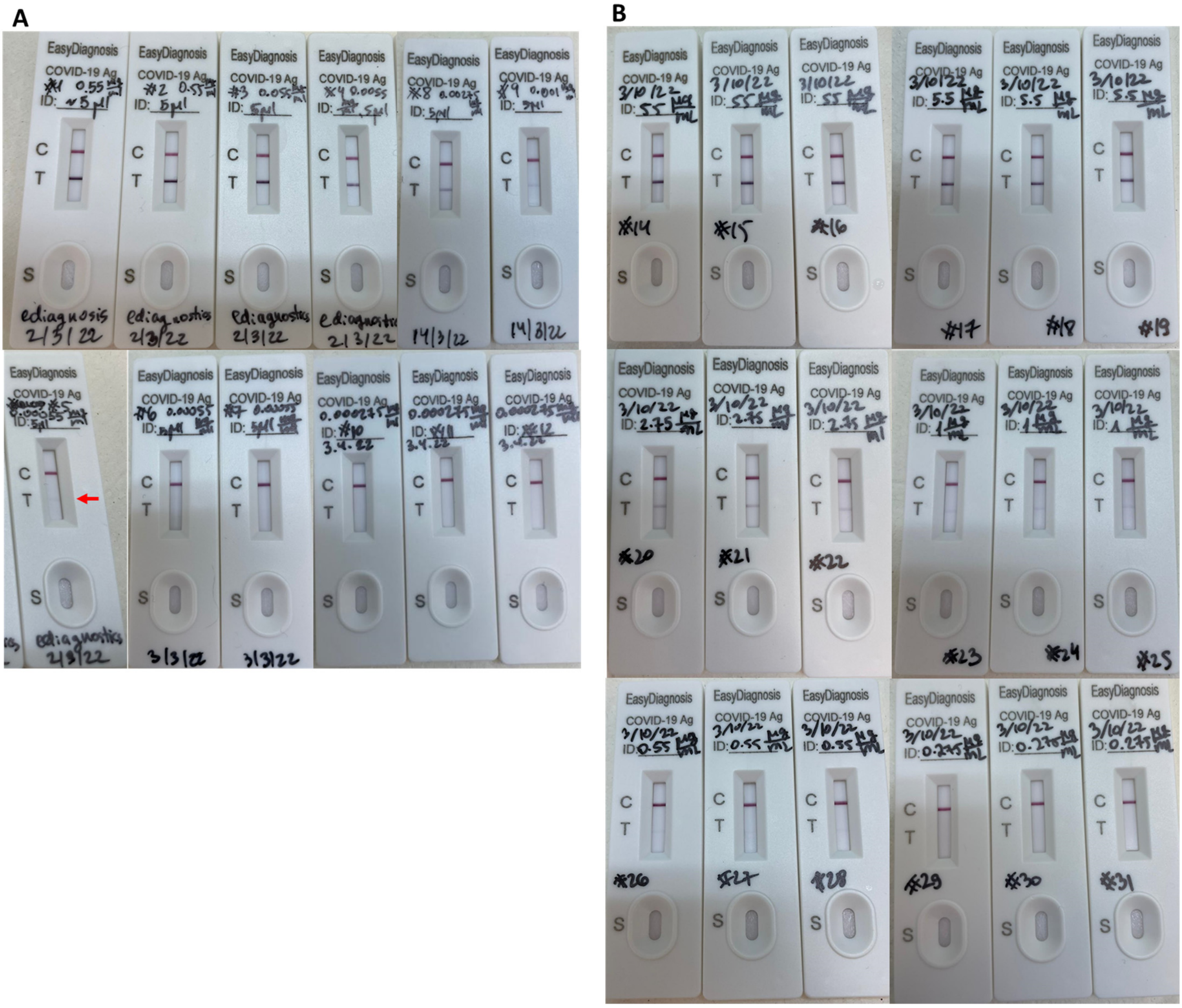

Figure S2. "Easy Diagnosis COVID-19(SARS-CoV-2) Antigen Test Kit" RATs after reaction with different concentrations of nucleocapsid protein at RH conditions of (**A**) 30% and (**B**) 60% . The lowest concentration that showed positive result, in all replicates at RH 60%, is 0.55 μg mL^{-1}; only one out of three RATs showed a weak T-line (red arrow) at RH 30%.

A

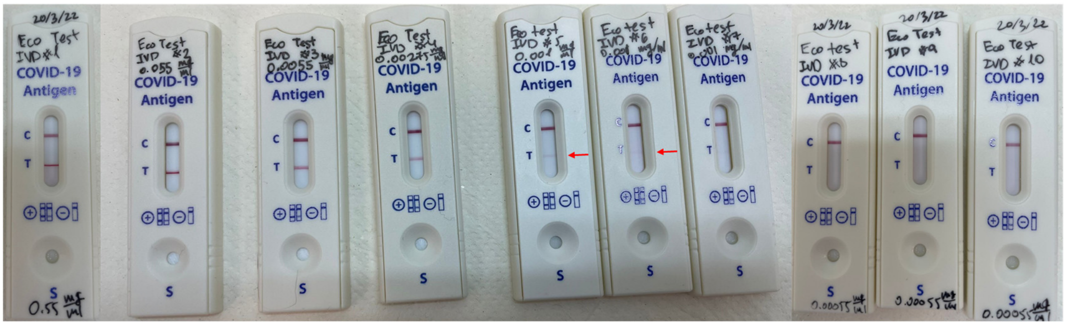

B

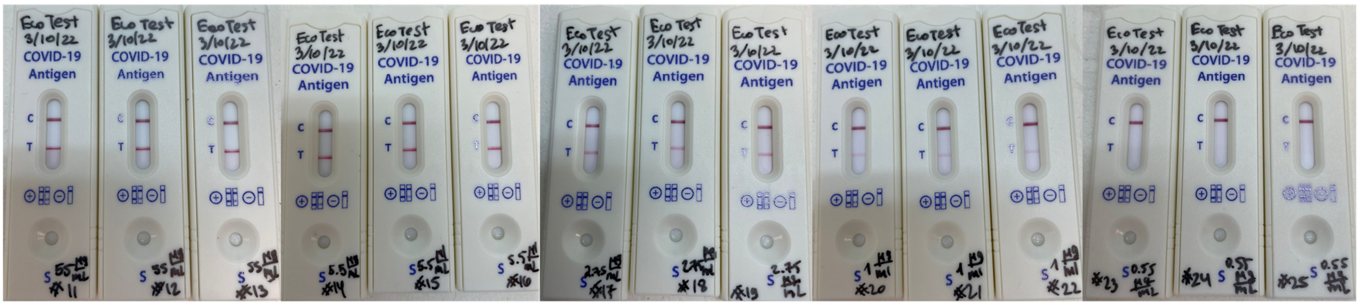

Figure S3. "EcoTest COVID-19 TO-GO" RATs after reaction with different concentrations of nucleocapsid protein. The lowest concentration that showed positive result is 1 µg mL^{-1} at RH conditions of (**A**) 30% and (**B**) 60%; Two out of three RATs showed a weak T-line (red arrows) at RH 30% and all replicates at 60%.

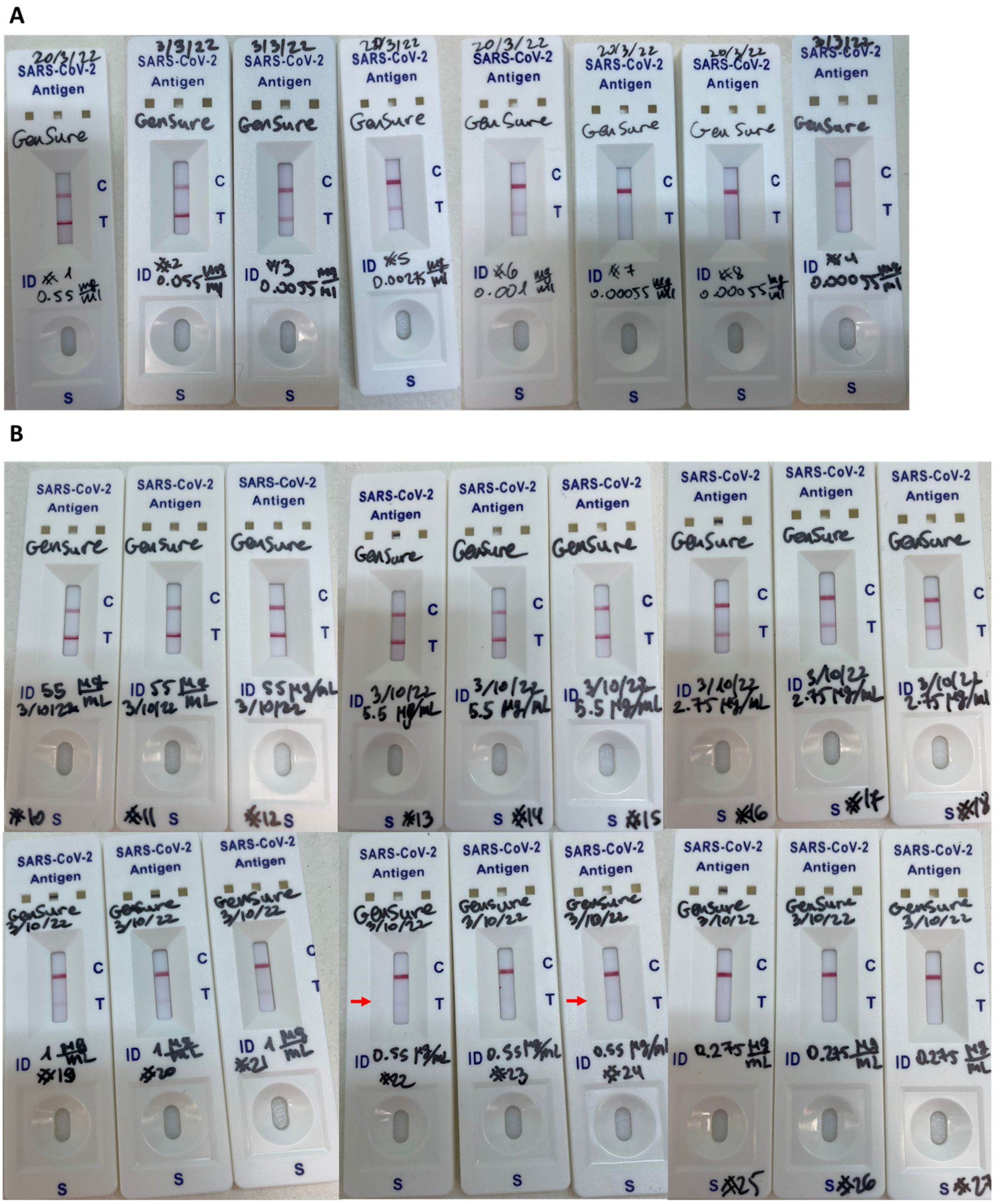

Figure S4. "GenSure COVID-19 Antigen Rapid Test Kit" RATs after reaction with different concentrations of nucleocapsid protein at RH conditions of (**A**) 30% and (**B**) 60%. The lowest concentration that showed a positive result is 0.55 µg mL^{-1} at RH 60%; two out of three tests showed positive results (red arrows).

A

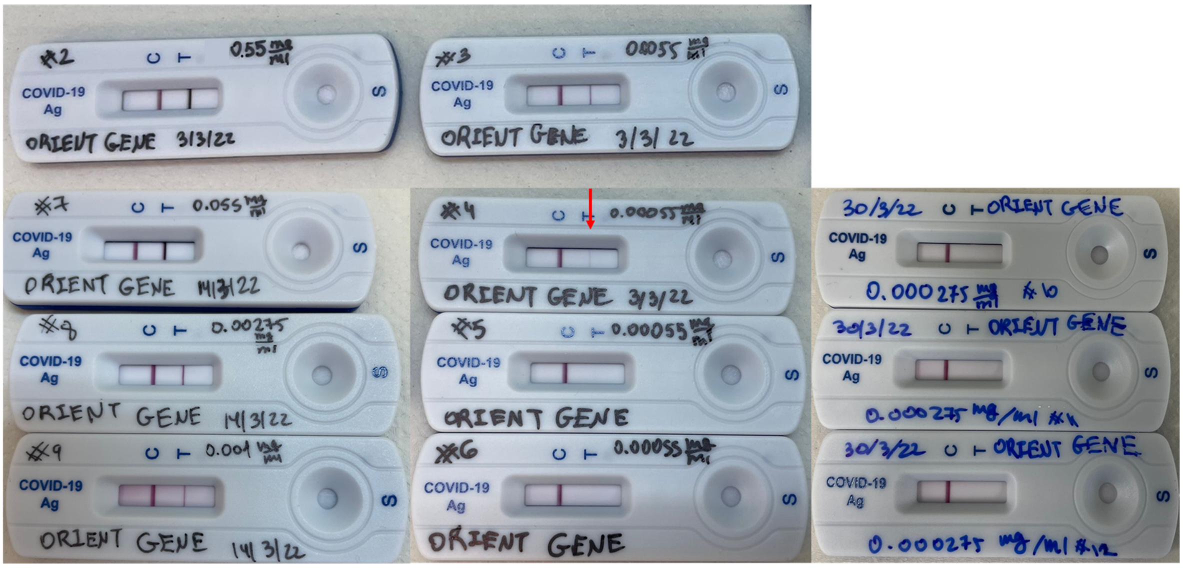

B

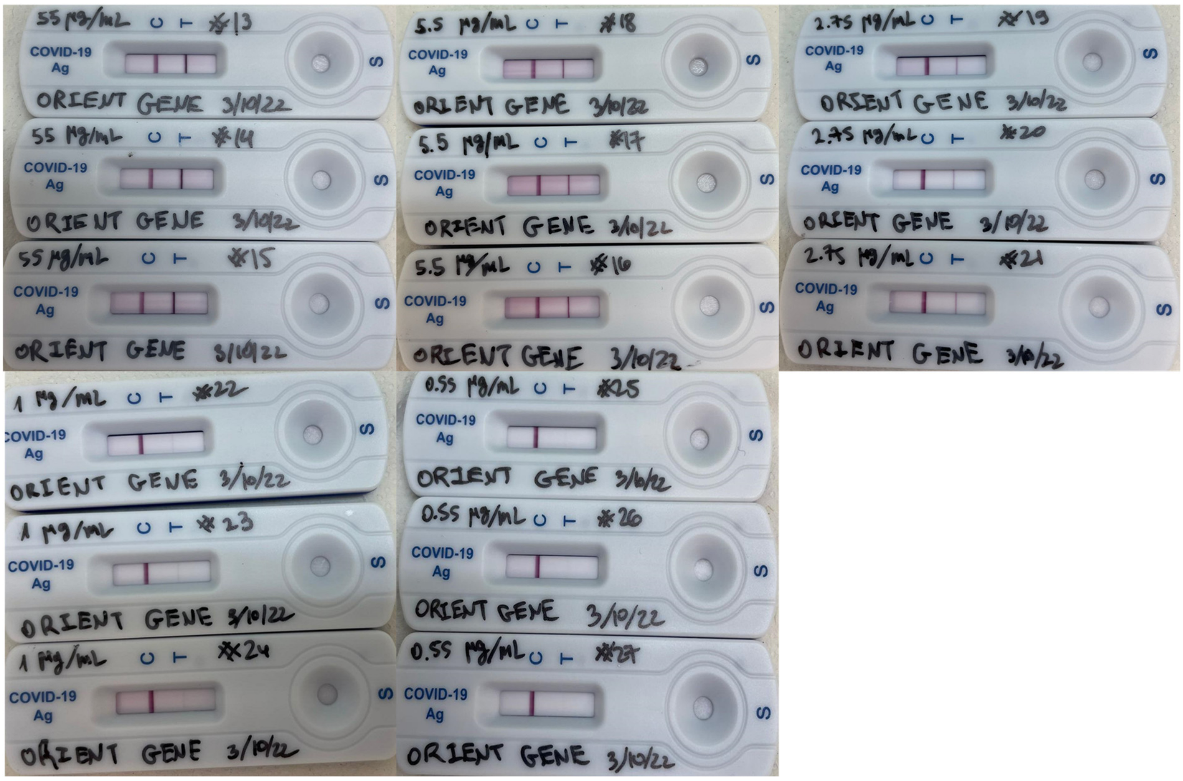

Figure S5. "Orient Gene Rapid COVID-19 (Antigen) Self-Test" RATs after reaction with different concentrations of nucleocapsid protein at RH conditions of (**A**) 30% and (**B**) 60%. The lowest concentration that showed positive result is 1 μg mL^{-1} at RH 60%. Only one out of three RATs showed a weak T-line (red arrow) using a concentration of 0.55 μg mL^{-1}, RH 30%.

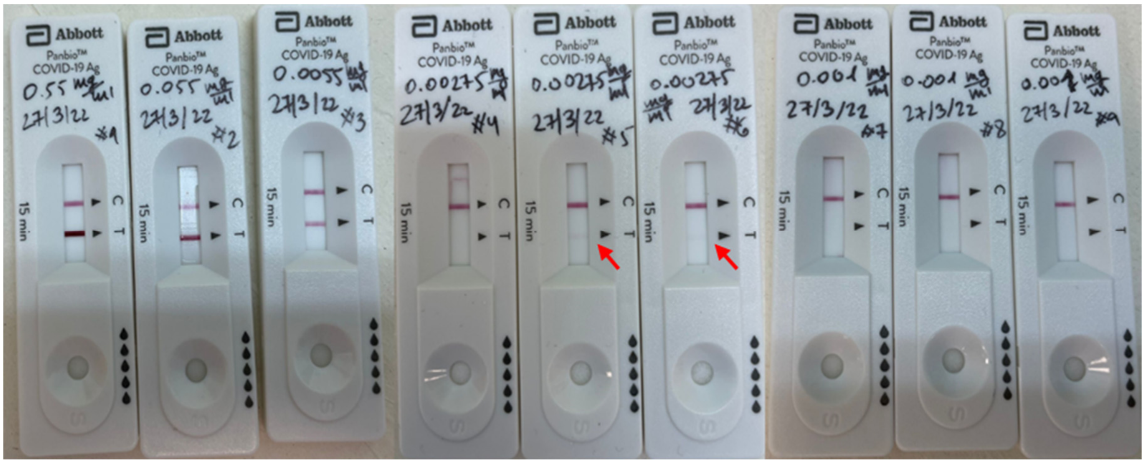

Figure S6. "Panbio COVID-19 Antigen Self-Test" RATs after reaction with different concentrations of nucleocapsid protein at RH 30%. The lowest concentration that showed positive result is 2.75 μg mL^{-1}; Two out of three RATs showed a T-line (red arrows).

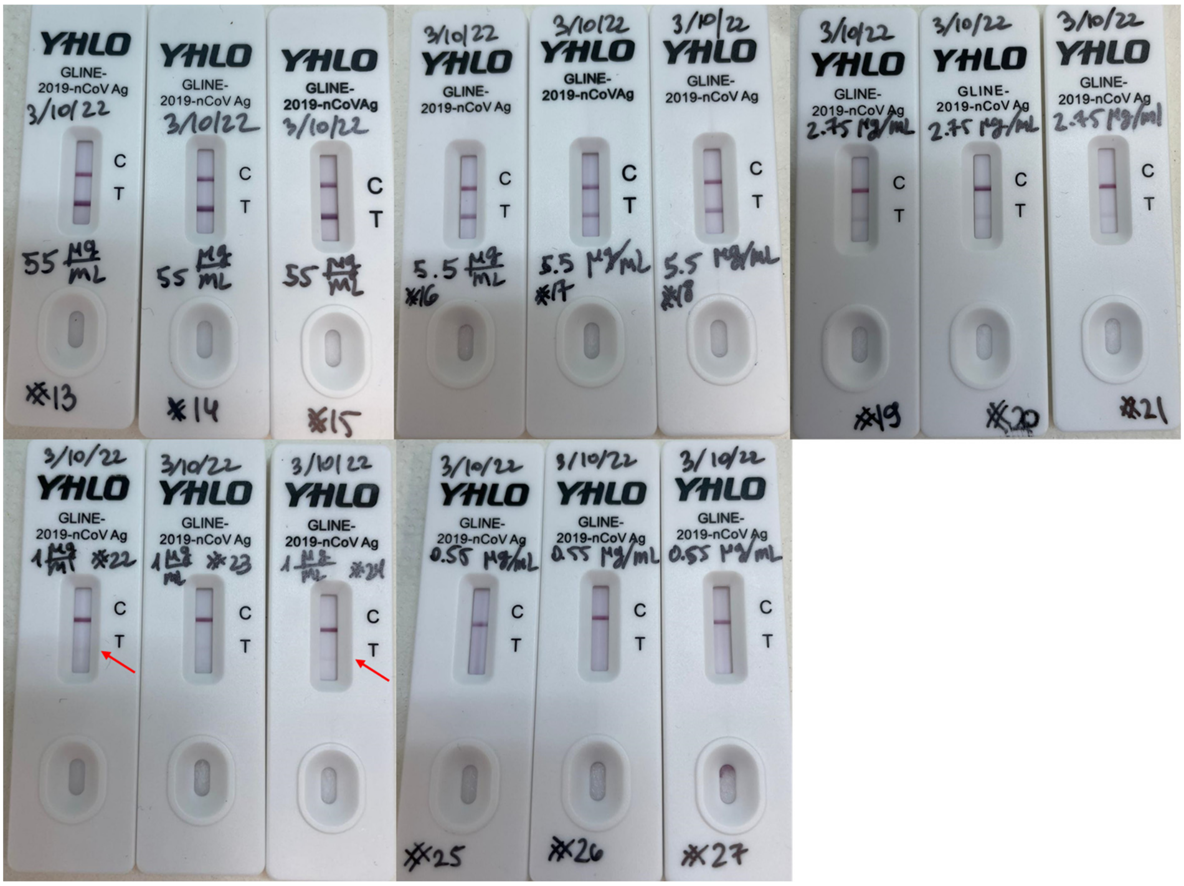

Figure S7. "YHLO GLINE-2019-nCoV Ag for self-testing" RATs after reaction with different concentrations of nucleocapsid protein at RH 60%. The lowest concentration that showed positive result is 1 μg mL^{-1}; Two out of three RATs showed a weak T-line (red arrow).

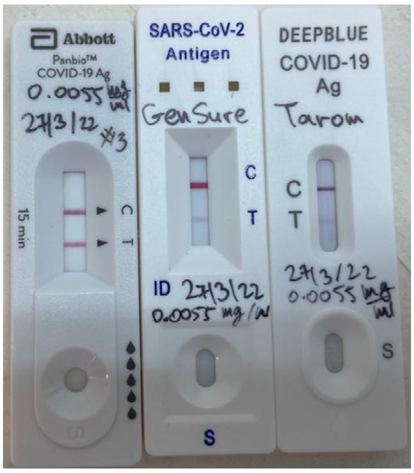

Figure S8. Three different RATs after reaction with 5.5 μg mL^{-1} nucleocapsid concentration on the same day. The T-line is the least prominent in the case of "Deepblue COVID-19 (SARS-CoV-2) Antigen Test Kit (colloidal gold)" RAT, intermediate intensity of the T-line observed in the case of "GenSure COVID-19 Antigen Rapid Test Kit" RAT, and the "Panbio COVID-19 Antigen Self-Test" RAT showed the most prominent T-line.
